# Engineered Schwann Cell-Based Therapies for Injury Peripheral Nerve Reconstruction

**DOI:** 10.3389/fncel.2022.865266

**Published:** 2022-05-06

**Authors:** Qisong Su, Moussa Ide Nasser, Jiaming He, Gang Deng, Qing Ouyang, Donglin Zhuang, Yuzhi Deng, Haoyun Hu, Nanbo Liu, Zhetao Li, Ping Zhu, Ge Li

**Affiliations:** ^1^Medical Research Center, Guangdong Provincial People’s Hospital, Guangdong Academy of Medical Sciences, Guangzhou, China; ^2^Guangdong Provincial People’s Hospital, Guangdong Cardiovascular Institute, Guangzhou, China; ^3^School of Biology and Biological Engineering, South China University of Technology, Guangzhou, China; ^4^School of Basic Medical Science, Shandong University, Jinan, China; ^5^School of Medicine, South China University of Technology, Guangzhou, China; ^6^Fuwai Hospital, Chinese Academy of Medical Sciences and Peking Union Medical College, Beijing, China; ^7^The First Clinical College, Guangdong Medical University, Zhanjiang, China; ^8^The Second School of Clinical Medicine, Southern Medical University, Guangzhou, China; ^9^Guangdong Provincial Key Laboratory of Structural Heart Disease, Guangzhou, China

**Keywords:** Schwann cells, peripheral nervous system, transcriptional regulators, tissue-engineered nerve graft, directed reprogramming

## Abstract

Compared with the central nervous system, the adult peripheral nervous system possesses a remarkable regenerative capacity, which is due to the strong plasticity of Schwann cells (SCs) in peripheral nerves. After peripheral nervous injury, SCs de-differentiate and transform into repair phenotypes, and play a critical role in axonal regeneration, myelin formation, and clearance of axonal and myelin debris. In view of the limited self-repair capability of SCs for long segment defects of peripheral nerve defects, it is of great clinical value to supplement SCs in necrotic areas through gene modification or stem cell transplantation or to construct tissue-engineered nerve combined with bioactive scaffolds to repair such tissue defects. Based on the developmental lineage of SCs and the gene regulation network after peripheral nerve injury (PNI), this review summarizes the possibility of using SCs constructed by the latest gene modification technology to repair PNI. The therapeutic effects of tissue-engineered nerve constructed by materials combined with Schwann cells resembles autologous transplantation, which is the gold standard for PNI repair. Therefore, this review generalizes the research progress of biomaterials combined with Schwann cells for PNI repair. Based on the difficulty of donor sources, this review also discusses the potential of “unlimited” provision of pluripotent stem cells capable of directing differentiation or transforming existing somatic cells into induced SCs. The summary of these concepts and therapeutic strategies makes it possible for SCs to be used more effectively in the repair of PNI.

## Introduction

Mechanical trauma, for example, as sustained in an industrial or traffic accident, is the most frequent cause of peripheral nervous system injury (Faroni et al., 2015). Approximately five million peripheral nerve injuries occur each year with 500,000 surgical operations performed in the United States alone, generating $1.5 billion for the nerve repair industry (Meena et al., 2021). Peripheral nerve injury (PNI) may result in permanent motor and sensory disabilities, and most individuals cannot return to regular employment immediately after injury. Additionally, recovery is poor because of the limited therapeutic impact, which affects patients’ quality of life and imposes a significant economic burden on society and families (Slavin et al., 2021). In contrast to the central nervous system (CNS), the adult peripheral nervous system (PNS) maintains considerable regenerative capacity even after serious injury (Chen et al., 2007). The considerable regeneration capability of the PNS is demonstrated by the capability of injured peripheral axons to regenerate and target their destinations, a process facilitated by the high plasticity of Schwann cells (SCs) in the PNS (Kim et al., 2013; Jessen et al., 2015).

**FIGURE 1:**
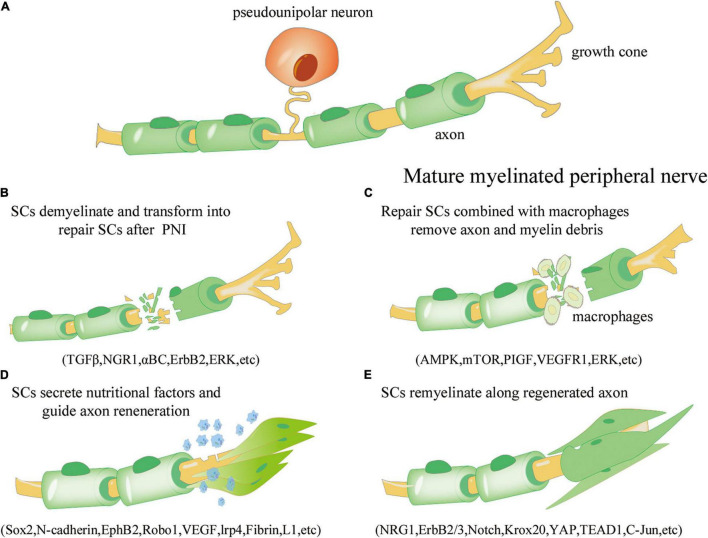
Overview of the repair process of Schwann cells (SCs) after peripheral nerve injury. **(A)** Under normal physiological conditions, mature myelinated peripheral nerve processes (yellow) are surrounded by the myelin sheath formed by SCs (green). **(B)** Axonal rupture and myelin disintegration due to peripheral nerve injury. SCs are activated, dedifferentiate, and transform into the repair phenotype. The involved signaling pathways include transforming growth factor beta (TGF-β), neuroregulin 1 (NGR1), αBC transporter, ErbB2, and extracellular signal receptor-kinase (ERK). **(C)** Repair SCs proliferate and fill the empty endoneurial canal, working with macrophages to remove axonal and myelin-derived debris; the involved signaling pathways include AMP activated protein kinase (AMPK), mammalian target of rapamycin (mTOR), placental growth factor (PIGF), vascular endothelial growth factor receptor 1 (VEGFR1), and ERK. **(D)** SCs are arranged along axons to form Büngner bands and secrete neurotrophic factors to improve the microenvironment and guide axon regeneration; the involved regulators include Sox1, N-cadherin, EphB2, Robo1, VEGF, lrp4, Fibrin, and L1. **(E)** SCs are re-myelinated along the regenerated axons to promote neural information transmission; the involved regulators include NRG1, ErbB2/3, Notch, Krox20, yes-associated protein (YAP), transcriptional enhance factor domain transcription factor 4 (TEAD1), and c-Jun.

SCs are a critical component of the PNS. Nerves in the periphery are composed of myelinated and unmyelinated fibers. Myelin sheaths, axons, and nerve membranes comprise nerve fibers. The larger axons establish a 1:1 connection with myelinated SCs, whereas the non-myelinated SCs bundle the smaller axons to form Remak bundles (Napoli et al., 2012). Peripheral nerve fibroblasts subsequently facilitate the formation of axon group bundles enclosed by SCs, and larger nerves consist of many bundles wrapped in the epineurium. Primary injury to these neural structures is often induced by direct forces, such as abrupt extension, tears, or compression, and the subsequent vascular ischemia exacerbates the primary injury, resulting in secondary injury (Sullivan et al., 2016). Within 12–24 h of PNS injury, calcium influx activates proteases, resulting in cytoskeleton rupture, axon membrane breakdown, and myelin degradation within 2 days (Schlaepfer and Bunge, 1973; Stoll et al., 1989). SCs dedifferentiate into a repair phenotype as a result of axonal breakdown and myelinolysis (Hall, 2005; Arthur-Farraj et al., 2017). Not only can repair SCs contribute to the clearance of axon and myelin-derived debris but they may also multiply to fill the empty endoneurial tube and create a strip-like column in the basal layer, called Büngner bands (Li et al., 2005; Murinson et al., 2005; Gomez-Sanchez et al., 2015). SCs in Büngner bands release neurotrophic factors that nourish injured neurons and promote axonal regeneration and myelination (Madduri and Gander, 2010; Jessen and Mirsky, 2016). The healing of PNS injury by SCs is outlined in Figure 1. To summarize, SCs are essential for the healing process after PNI. It is critical to study the many identities of SCs that rely on various important chemicals throughout the development, injury, and repair of the PNS to achieve more effective treatment methods for preventing or removing micro-environmental problems in myelination injury.

Given the critical role of SCs in the healing process of PNI, this article presents a comprehensive analysis of the major molecular regulatory networks of SCs involved in the treatment of PNI over the last decade to identify significant targets for increasing SC repair efficiency. Using existing genetic modification of SCs and induced Schwann-like cells (iSCs), their applications in the repair of PNI are summarized, and gene editing using cutting-edge technology to enhance the effect of SC repair possibilities is explored. An “unlimited” supply potential of pluripotent stem cell directional differentiation or conversion of available class cells to iSCs can occur and is explained in this review. The comprehensive review of these studies has enabled researchers to make new advancements from conventional SC transplantation treatment to SC engineering and manufacture and to provide new ideas for stimulating related research.

## Developmental Lineage and Post-Injury Gene Regulation Network of Schwann Cells

### Schwann Cells Are Derived From Ectodermal Neural Crest Cells

**FIGURE 2:**
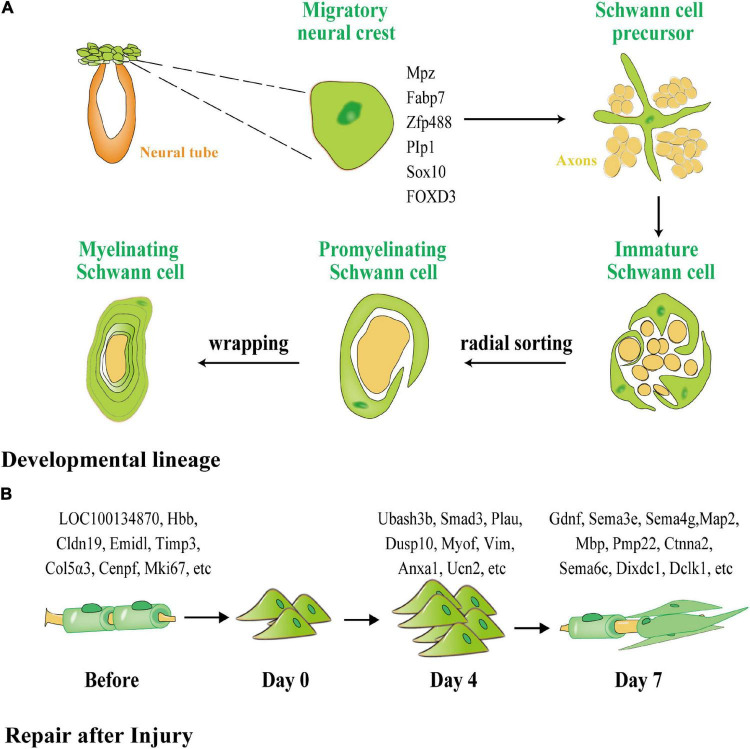
The different processes of myelination of SCs during development and after peripheral nerve injury (PNI). **(A)** SCs generated from neural crests create myelin sheaths in the PNS. Green represents glial cells and precursors, and orange represents the neural tube and yellow represents neurons and their axons. SCPs are neural crest cells that detach from the neural tube and migrate along the nascent peripheral axons, expressing the early glial markers Mpz, Fabp7, Zfp488, Plp1, Sox10, and forkhead box D3 FOXD3. SCPs differentiate into immature SCs, which, *via* cytoplasmic extension, enclose the axon bundle. Finally, radial sorting is used to choose a single axon for wrapping. SCs that have been myelinated enclosed axon segments, forming myelin sheaths. **(B)** Dedifferentiation and remyelination of SCs after PNI. Normal SCs form a myelin sheath around axons with one of four phenotypes: subtype 1 expressing LOC100134871 and Hbb, subtype 2 expressing Cldn19 and Emid1, subtype 3 expressing Timp3 and Col5a3, and subtype 4 expressing Cenpf and Mki67. After PNI, SCs transform into a homogenous phenotype and participate in proliferation and phagocytosis. On the fourth day after injury, 776 lncRNAs were unique to the proliferation of SCs. After day 7, 317 lncRNAs were unique to the remyelination of SCs.

SCs are derived from neural crest cells (NCCs), which ultimately create myelin sheaths through two intermediate stages: (1) Schwann cell precursors (SCPs) and (2) immature SCs, which provide nutritional support for the development of peripheral nerve axons (Figure 2A). NCCs develop throughout the PNS growth process by forming new peripheral axons with strong proliferative capabilities and high pluripotency (Muppirala et al., 2021). The first stable branch separates the sensory lineage from the common progenitor cells of the autonomic and mesenchymal branches during the migration of NCCs. The second stable branch divides the production of autonomic neurons, and the remaining branches can be attributed to glial differentiation owing to their expression of the transcription markers for early glial cells (*Mpz, Fabp7, Zfp488, Plp1, Sox10*) and transcriptional markers of SCPs (Soldatov et al., 2019). SCP migration starts with the formation of a basal layer (BL) and the process of wrapping the axons during the juvenile SC stage. When immature SCs are wrapped in a 1:1 manner throughout the radial separation phase, proliferation of the immature SCS slows, and definitive differentiation begins. This phase of differentiation is controlled by the BL, which facilitates an increase in the expression of myelination genes. After differentiation, the juvenile SC skeleton progressively grows until its membrane wraps around the axon and produces a myelin sheath. Following the completion of the package, myelin is compressed to create a mature myelin sheath. Stable and mature SCs are then formed through active signaling pathways.

SCs exhibit distinct gene regulatory networks after PNI when compared with SC development (Figure 2B). According to Chen et al. (2021), the cells in normal and injured peripheral nerves may be functionally classified as SCs, neurofibroblasts, immune cells, and vascular-related cells. SCs and neurofibroblasts are critical for the regeneration of peripheral nerves. Zhang et al. (2021) discovered four distinct phenotypes in SCs in newborn rats using single-cell transcriptome analysis, namely, SC subtype 1 resembling connective tissue cells expressing *LOC100134871* and *Hbb*, and highly mature SC subtype 2, expressing *Cldn19* and *Emid1*. *Timp3* and *Col5a3* were found to be expressed in subtype 3, which is involved in tissue development and differentiation into other SC subtypes, whereas *Cenpf* and *Mki67* were found to be expressed in subtype 4, which is related to cell division, peripheral nerve development, and regeneration. Zhang et al. (2021) used second-generation sequencing to perform deep sequencing in rats on days 4 and 7 (D4 and D7) respectively after sciatic nerve injury, and discovered that 776 long non-coding RNAs (lncRNAs), such as *Ubash3b, Smad3, Plau*, and others, are unique to D4. Most of these RNAs are related to wound healing, phosphatase binding, and the mitogen-activated protein kinase (MAPK) signaling cascade. *Gdnf, Sema3e, Sema4g*, and others were found on D7 and appear to be associated with axon regeneration and cell cycle. Throughout development and repair, these diverse expression regulatory networks indicate that separate neural regulation methods for differentiation and induction of iSCs and the mobilization of repair following injury can be used.

### Key Signaling Pathways Involved in Migration, Proliferation, and Axon Guidance of Schwann Cells After Peripheral Nerve Injury

Following PNI, mature SCs may undergo de-differentiation through various pathways, migrate to the wounded area, stimulate nerve remyelination, and play a role in directing axon regeneration. Wang et al. (2012) investigated the intrinsic migration characteristics of SCs using single-cell migration experiments and discovered that de-differentiated SCs first develop long protuberances, during which the nucleus was shown to move forward to the front of the cell and then retract to the back of the protuberances after which the protuberances extended again. Numerous studies have shown links between transforming growth factor beta (TGF-β), Slit-Robo, ErbB2 receptor, Hippo and Notch, extracellular signal -related kinase (ERK), and vascular endothelial growth factor (VEGF) signaling pathways and migration, proliferation, and axon guidance of SCs after PNI (Table 1).

**TABLE 1:** Key signaling pathways involved in migration, proliferation and axon guidance of SCs after PNI.

Signaling pathways	Factors	Function	References
TGF-β	TGF-β, N-cadherin, EphB2, SOX2	Dedifferentiation, sorting and migration of SCs, axon regeneration	Parrinello et al., 2010; Clements et al., 2017
Slit-Robo	Slit,Slit2,Slit3	Axon pathfinding, migration of SCs	Blockus and Chédotal, 2016; Dun et al., 2019
ErbB2 Receptor	NRG1, αBC	Proliferation of SCs, axon wrapping and myelination	Garratt et al., 2000; Hendry et al., 2016; Lim et al., 2017
Notch and Hippo	Notch, NICD, Krox20, TEAD1, YAP, TAZ	Proliferation and myelination of SCs	Ghislain and Charnay, 2006; Woodhoo et al., 2009; Hansen et al., 2015; Grove et al., 2017
ERK	Raf, fibrin, ERK, c-Jun	Dedifferentiation and myelination of SCs.	Akassoglou et al., 2002; Harrisingh et al., 2004; Syed et al., 2010; Napoli et al., 2012
VEGF	HIF-1α, VEGF	Proliferation and migration of SCs	Sondell et al., 1999; Cattin et al., 2015

*TGF-β, transforming growth factor-β; NRG1, Neuregulin 1; αBC, AlphaB-crystallin; Notch, Neurogenic locus Notch homolog protein; HIF-1α, hypoxia inducible factor −1α; VEGF, vascular endothelial growth factor.*

**FIGURE 3:**
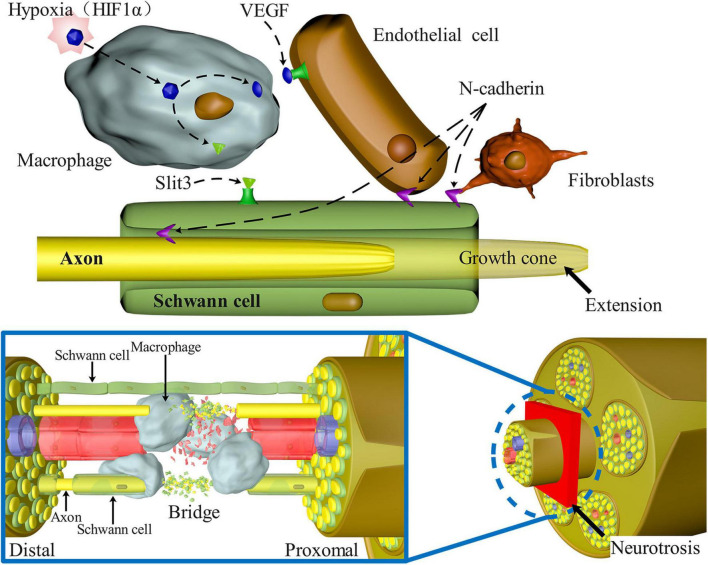
Signaling pathways involved in SC migration, proliferation, and axon guidance in nerve bridges after peripheral nerve injury. N-Cadherin increases the adhesion between SCs, fibroblasts, and endothelial cells, causing SCs to migrate to the outside of the nerve stump and guiding axons to reorient growth at the injury site. Macrophages enter the nerve space and secrete Slit3, which binds with SCs expressing Robo1 to control the formation of correct nerve bridges and promote SC migration and axon guidance. Macrophages in the nerve bridges detect hypoxia and enhance the amount of VEGF-induced blood vessel regeneration, alleviating hypoxia, and acting as an important medium to guide axon regeneration.

Figure 3 depicts a schematic representation of the many signaling pathways involved in the migration of SCs after PNS injury.

## Targeting Genetically Engineered Schwann Cells to Improve the Repair Efficiency of Peripheral Nerve Injury

Numerous studies have shown that SCs are capable of successfully facilitating PNI repair. Through genetic engineering, the native genes of SCs may be up- or down-regulated, and the natural products required for peripheral nerve regeneration can be generated in a controlled manner. SCs may be genetically modified using various tools and methods that introduce specific genes or sequences into the genome, such as viral vectors, non-viral transfection systems, and clustered regularly spaced short palindromic repeats (CRISPR)/Cas9 gene-editing approaches.

### Intervention With Histone Deacetylase Affects the Maturation and Myelination of Schwann Cells Through Notch-Hey2 Signaling

Histone deacetylase (HDAC) is a significant epigenetic regulator of the Notch–Hey2 signaling pathway, which is required for myelination in the peripheral nervous system. Two groups of HDACs, HDAC1 and 2, both of which are involved in the myelination of SCs and HDAC3, which is involved in maintaining myelin homeostasis have been found. Jacob et al. (2011) used lentiviral transfection to selectively knockdown HDAC1 and 2 in SCs, a process leading to a substantial decrease in the expression of SOX10 and Krox20, the main transcriptional regulators of myelination in SCs. HDAC1 and 2 have been shown to have distinct main roles in SCs, with HDAC2 cooperating with Sox10 to initiate the myelination transcriptional process. Concurrently, HDAC1 regulates SC survival by controlling the amount of active β-catenin.

Likewise, Chen et al. (2011) discovered that the absence of HDAC1 and 2 prevented the NF-κB protein complex from binding to p300 and HDAC1/2, resulting in significant acetylation of NF-κB p65, reduction in the positive regulators of myelin development, and activation of differentiation inhibitors. In addition, development of SCs stalled during the immature stage. Interestingly, Brügger et al. (2017) discovered that early treatment with HDAC1/2 inhibitors caused an increase in the production of repair SCs and improvement in axonal regeneration and functional recovery but also led to impaired myelin regeneration. This process raises the possibility of adjusting the expression period of HDAC1 and 2 in SCs after injury during clinical transformation to improve peripheral nerve regeneration. Rosenberg et al. (2018) demonstrated the unique effects of HDAC3, another class of protein deacetylases involved in the Notch-Hey2 signaling pathway, on SCs *via* gene knockout. These authors demonstrated that although HDAC3 was not found to be necessary for the formation of myelinated SCs, once myelinated SCs are formed, HDAC3 appears to be required for their stability. He et al. (2018) discovered that HDAC3 caused suppression of the neuroregulin/phosphoinositide 3-kinase/protein kinase B (NRG1/PI3K/Akt) signaling pathway and its downstream myelination process, ensuring correct myelination and preventing peripheral myelin expansion *in vivo*.

Additionally, HDAC3 may bind the p300 histone acetyltransferase and control the expression of the transcriptional enhance factor domain transcription factor 4 (TEAD4) gene thus activating the inhibitory network of myelin development. Zinc-finger e-box-binding Homeobox 2 (Zeb2) is another important regulator of the Notch–Hey2 signaling cascade. Wu et al. (2016) generated Zeb2-defective SCs by non-viral transfection and discovered that Zeb2 regulated the start of SC development by recruiting the co-inhibitory histone deacetylases, HDAC1 and 2, and nucleosome remodeling and histone deacetylation (HDAC1/2-NuRD). Hey2 appears to limit differentiation of SCs by preventing differentiation and promoting their proliferation, whereas Zeb2 seems to promote the maturation and myelination of SCs by directly inhibiting Hey2 and regulating the Notch–Hey2 signaling pathway.

### Intervention of Mitogen-Activated Protein Kinase and Hippo Signaling Pathways to Promote the Proliferation and Myelin Regeneration of Schwann Cells

Moesin-ezrin-radixin-like protein (Merlin) tumor suppressors contribute to cell signaling, contact-mediated proliferation, and tumorigenesis. Loss of Merlin was found to disrupt the MAPK and Hippo signaling pathways, leading to enhanced activity of the Hippo effectors, yes-associated protein 1 and (YAP) and related protein, TAZ (Ammoun et al., 2008; Cooper and Giancotti, 2014). Merlin deficiency results in a significant increase in SC proliferation, macrophage infiltration, severe injury to axons and myelin regeneration, decreased induction of c-Jun, dysregulation of the MAPK signaling pathway, and activation of the Hippo signaling pathway following injury (Mindos et al., 2017). Previous studies have demonstrated that silencing YAP in SCs could functionally restore both axon and myelin sheath regeneration caused by Merlin loss and restore the expression of c-Jun and neurotrophic factors in addition to axon regeneration and functional recovery following injury. Deng and coworkers (Deng et al., 2017) showed that activating the Hippo signaling pathway led to an increase in SC proliferation, while TAZ inhibited the Gnas gene, which produces the GαS protein. Gnas deficiency resulted in a dramatic increase in the proliferation of SCs. Consequently, it was hypothesized that Gnas control may aid in limiting SC growth. However, Grove et al. (2020) showed that knock down of YAP/TAZ does not impair the proliferation of SCs or enhance transition of SCs into repair SCs. However, axon sorting and myelin degeneration are impaired in the absence of YAP/TAZ (Poitelon et al., 2016; Grove et al., 2017, 2020). This process occurs because growing SCs need YAP/TAZ to enter the S phase and generate sufficient SCs for correct axon sorting. Concurrently, YAP/TAZ is needed for TEAD1 to activate Krox20, which regulates myelination (Grove et al., 2017).

### Activation of c-Jun and Overexpression of Neurotrophic Factors in Schwann Cells Regulate Neural Regeneration Potential

Activation of the transcription factor c-Jun in SCs acts as a global regulator of Waller’s degeneration and regulates the expression of nutrient factors, adhesion molecules, regeneration locus formation, and myelin clearance. In addition to activating repair programs in SCs, this factor contributes to generating cells specifically designed for regeneration, thereby regulating the regeneration potential of peripheral nerves (Arthur-Farraj et al., 2012). c-Jun deficiency results in the development of defective repair SCs, impairs functional recovery, and results in neuronal death. c-Jun overexpression can effectively lead to an increase in expression and secretion of a variety of neurotrophic factors (NFs), including glial cell line-derived neurotrophic factor (GDNF), brain-derived neurotrophic factor (BDNF), artemin, leukemia inhibitory factor (LIF), and nerve growth factor (NGF), thereby promoting the migration of SCs. In addition, c-Jun overexpression enhances local proliferation of SCs and neurite development in the presence of axons (Huang L. et al., 2015). Lackington and colleagues (Lackington et al., 2018) created plasmid-carrying nanoparticles and transfected SCs to overexpress nerve growth factor (NGF), glial cell line-derived neurotrophic factor (GDNF), and c-Jun. A comparative study revealed that all three types of cells could promote neurite development; however, SCs overexpressing c-Jun had the highest capacity for cell regeneration.

SCs produced an increase in the expression of several axon-regenerating NFs in response to PNI (Hammarberg et al., 1996; Höke et al., 2000; Eggers et al., 2010). By targeting NFs expression with advanced genetic engineering methods, SC repair efficiency after injury could be substantially increased. Shakhbazau et al. (2012) transfected SCs with either NGF or GDNF and discovered that SCs overexpressing NGF enhanced axonal regeneration to a much greater degree. However, NGF and GDNF overexpression may have various consequences on sensory and motor regenerating neurons. The increase in NGF and GDNF levels did not affect the number of regenerated sensory neurons at 1 cm from the distal end of the injury at 4 weeks after injury. GDNF overexpression was found to promote long-distance axon development and nerve re-innervation of the target muscle in motor neurons, whereas NGF had little impact on these parameters (Tannemaat et al., 2008). However, axon regeneration is sluggish and requires considerable time to complete. Marquardt et al. (2015) developed a system for controlling GDNF release over time and in space *via* lentiviral transfection of SCs and combining this system with biomaterials. They discovered that GDNF overexpression at 4–8 weeks promoted axon regeneration and muscle mass recovery, but that too short a delivery time (4 weeks) prevented axon extension and that too long a delivery time (> 8 weeks) could also result in axon regeneration failure.

Fibroblast growth factor 2 (FGF-2) is a neurotrophic factor produced by fibroblasts and SCs. This factor plays a critical role in SC proliferation and migration (Chen et al., 2010). FGF-2 subtype differences may have a profound impact on nerve regeneration. By comparing SCs overexpressing 18 kDa FGF-2 to SCs overexpressing 21/23 kDa FGF-2, Haastert et al. (2006) discovered that 18 kDa FGF-2 hinders the myelination of regenerated axons. However, 21/23 kDa FGF-2 enhanced the early recovery of sensory function and the myelination of long-distance axons in regenerated axons. Allodi et al. (2014) transplanted SCs overexpressing 18 kDa FGF-2 into a rat model of sciatic nerve injury and observed that nerve reinnervation of the hind limb muscles was accelerated and more obvious following transplantation. The number of motor and sensory neurons reaching the distal nerve increases at the end of follow-up, a process that is conducive to injury repair.

Studies have shown that neurotrophin-3 (NT-3) is a neurotrophic factor that helps reduce inflammatory responses and promotes cell survival and migration in poor microenvironments after injury (Randolph et al., 2007; Li et al., 2016, 2021a). Woolley et al. (2008) discovered that loss of NT-3 led to enhancement of caspase-3 protein production in SCs, suggesting that NT-3 is required for proper SC survival and differentiation. Additionally, NT-3 released by SCs at the axon–glial interface may act on axons and promote release of neuroregulatory proteins, which subsequently signal SCs to accelerate myelination. Zong et al. (2013) implanted SCs into a rat model of sciatic nerve injury. They found that SCs overexpressing NT-3 caused a decrease in motor neuron apoptosis in the injured sciatic nerve, accelerated nerve and axon regeneration, and enhanced the function of the injured nerve.

## Reconstruction of Peripheral Nerve Tissue Morphology and Function Based on a Combination of Schwann Cells and Biomaterials

**FIGURE 4:**
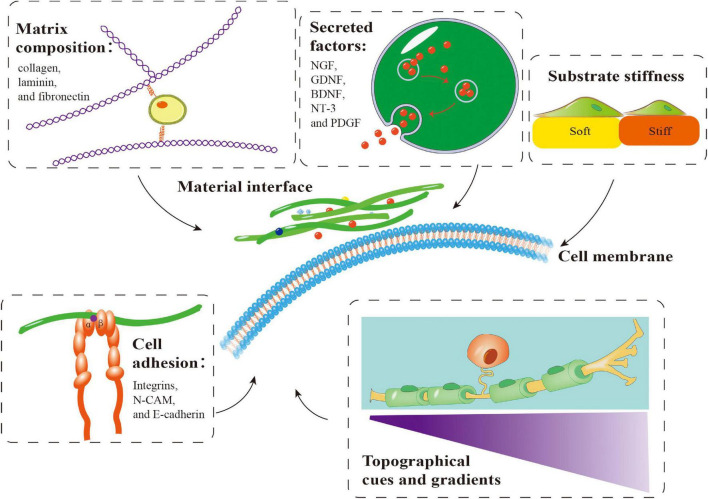
Important factors involved in the application of biomaterials in combination with Schwann cells (SCs) in peripheral nerve regeneration. Matrix components, secreted factors, cell adhesion, substrate stiffness and topographical cues and gradients influence the behavior of SCs on biomaterials, which is crucial for peripheral nerve regeneration. NGF, nerve growth factor; GDNF, glial-derived neurotrophic factor; BDNF, brain-derived neurotrophic factor; NT-3, neurotrophin-3; PDGF, platelet-derived growth factor.

Repair of PNI with a long-distance nerve deficit present a significant challenge. The gold standard for PNI healing has always been autologous nerve tissue transplantation. Autologous transplantation at the donor site may result in pathological alterations, such as sensory abnormalities, neuroma, infection, and other potential complications. Additionally, autografts are only available in a limited quantity (Lee and Wolfe, 2000). As a result, it is critical to create efficient nerve healing methods to circumvent autologous transplantation restrictions. Similarly, various biomaterials have been used to treat PNI. These biomaterials can provide favorable conditions for peripheral nerve regeneration by simulating the microenvironment through matrix components, secreted factors, cell adhesion, substrate stiffness, and topographical cues and gradients (Figure 4). These biomaterials may be roughly classified into natural and synthetic biomaterials, each with clear benefits. Synthetic biomaterials can adjust material properties to meet various repair needs, such as enhancement of cell adhesion and regulation of mechanical characteristics (Li et al., 2015; Mobasseri et al., 2015; Shahriari et al., 2017). In contrast to synthetic biomaterials, natural materials (such as autografts, allografts, and acellular grafts) generate non-toxic degradation products, possess intrinsic cell-binding domains, and can efficiently activate natural tissue remodeling and repair pathways (Bonnans et al., 2014; Nicolas et al., 2020).

### Natural Acellular Grafts and Schwann Cells

He et al. (2015) created a human acellular nerve graft (ANG) to replace autologous nerves and showed that ANG was safe and efficient for nerve deficits ranging in size from 1 to 5 cm. By obliterating antigenic biological components, ANGs, in comparison to allografts, can lead to a decrease in the immune responses. The impact of ANGs on long and massive nerve deficits, in contrast, is far from acceptable. As a result, Qiu et al. (2015) modified ANGs using the cartilage oligomeric matrix protein (COMP)-angiopoietin-1 (Ang1). COMP-Ang1 was found to enhance early neovascularization followed by rapid nerve regeneration, thus significantly increasing the effectiveness of ANGs in healing peripheral nerve lesions in clinical studies. Zhu et al. (2015) used Ginkgo biloba extract 761 (EGb 761) after ANG transplantation in rats with sciatic nerve injury. They discovered that ANG repair treated with EGb 761 showed the same effects on peripheral nerve regeneration and vascularization as autograft repair and was superior to ANG repair alone. However, ANGs are always deficient in the SCs required for nerve regeneration, limiting nerve regeneration in pure ANGs and rendering them inferior to autografts or allografts (Bunge, 1994; Whitlock et al., 2009). By transplanting SCs into ANGs, Jesuraj et al. (2014) discovered that SCs could enhance growth factor expression in ANGs and exhibit equivalent muscular strength and nerve fiber regeneration to allografts, thus boosting peripheral nerve regeneration and functional recovery.

In conclusion, ANG may facilitate more complete removal of cells, myelin sheaths, and other components associated with immune rejection from the grafts, thus lowering the risk of immunological rejection and increasing histocompatibility. In addition, ANGs preserve the nerve’s original structure and efficiently direct the regenerated axons to their target organs. Sun et al. (2020) showed that decellularization selectively removes axon-suppressing molecules, such as myelin associated glycoproteins and chondroitin sulfate proteoglycans present in normal nerves and retains axon-promoted extracellular matrix (ECM) proteins, including collagen IV and laminin. The preserved extracellular matrix components create a milieu similar to peripheral nerve tissue for SC adhesion and proliferation (Carriel et al., 2014). In their latest study, Wang et al. (2022) found that components and spatial organization of the extracellular matrix secreted by bone marrow MSCs closely resemble the acellular nerve and have stronger expression of factors related to nerve regeneration and lower immune responses; therefore, MSCs may be more likely to provide potential alternatives for clinical repair of peripheral nerve defects.

### Synthetic Neural Catheter and Swann Cells

The synthetic nerve-guided catheter (NGC) is another method of replacing autologous nerves. In contrast to allogeneic acellular nerve, NGCs have no donor restriction and are simpler to manufacture on a large scale. The tubular structure of the NGCs bridges the nerve stump and prevents the regenerated nerve from being influenced by the surrounding tissue while concurrently directing the regenerated axon to the distal stump properly. By creating a polyglycolic acid (PGA) multi-channel guidance scaffold, Hu et al. (2008) recruited and directed endogenous SCs to move along the PGA wire and form a cell column comparable with Büngner bands involved in PNI repair. However, nerve regeneration involves cell interactions, the extracellular matrix, and growth factors. Consequently, nerve injury repair methods have evolved from simple nerve catheters to sophisticated tissue engineering approaches that closely resemble the complex milieu seen in autografts. Chitosan has excellent biocompatibility, permeability, plasticity, and biodegradability, making it a suitable candidate material for the construction of artificial nerve grafts (Nawrotek et al., 2016). Chitosan-based nerve grafts mediate a novel molecular mechanism of nerve regeneration in that chitosan degrades into chitosan oligosaccharides following transplantation. This process was found to lead to a decrease in the expression of miR-327 in SCs and increase in CCL2 expression, thereby inducing macrophage infiltration, rebuilding of the microenvironment at the site of injury, and promotion of nerve regeneration (Zhao et al., 2017). Graphene oxide (GO) is a nanomaterial with extraordinary physical and chemical characteristics. Polycaprolactone (PCL) is a biocompatible polymer scaffold with an appropriate hardness. The nano-scaffolds prepared using GO and PCL are beneficial for the proliferation, survival, adhesion, and maintenance of neural properties in SCs. They can effectively promote functional and morphological recovery of peripheral nerves, which is promising for tissue engineering (Qian et al., 2018). Zhang et al. (2020) demonstrated that micropatterning on the inner wall of NGCs constructed with GO was conducive for promoting SCs to secrete neurotrophic factors, such as NGF and BDNF, guiding the direction of axons, inducing macrophage M2 differentiation, and promoting nerve regeneration and the recovery of normal functions. Fadia et al. (2020) created PCL nerve conduits implanted with GDNF microspheres and discovered that they could cause a substantial increase in SC proliferation and nerve conduction when compared with autografts but had no meaningful effect on functional function recovery.

Manipulating the microstructure and mechanical characteristics of biological scaffold materials can alter the differentiation efficiency of Schwann cell-like cells. Electrospinning fibers can be arranged, stacked, or folded to form organized arrays or layered structure and effectively direct axon regeneration (Xie et al., 2010; Huang C. et al., 2015; Xue et al., 2017a). Furthermore, they can efficiently stimulate the development of mesenchymal stem cells (MSCs) into SCs and control the morphology and arrangement of source cells (Xue et al., 2017b). Hu et al. (2020) showed that the combination of amine-functionalized multi-walled carbon nanotubes (MWCNT) with PCL and gelatin and preparation of directional or random conductive nanofibers by electrostatic spinning could lead to a significant improvement in the differentiation efficiency of BMSCs into SCs. This process could also promote axon regrowth in the peripheral nervous system. This research indicates a good solution to address the difficulties associated with the source of SCs and required improvements in the differentiation and functionalization of stem cells to SCs. Thermally-induced phase separation (TIPS) technology has the potential to influence the efficiency of MSCs to transdifferentiate into SC-like phenotypes by causing alterations in the microstructure and mechanical properties of the nanofibers and macroporous and ladder-like structures found in gelatin-based three-dimensional (3D) tubes (Ma and Zhang, 1999; Zeng et al., 2014). Uz et al. (2017) demonstrated that the conduit structure with macroporous and ladder 3D structures could enhance the attachment, proliferation, and diffusion of MSCs to create an interconnected cell network with many living cells and achieve an optimal microenvironment for the transformation of MSCs into SC-like phenotypes. Wu et al. (2020) synthesized composite nanofiber yarns (NYs) from poly(p-dioxanone) (PPDO) and carbon nanotubes (CNT). CNTs are added to NYs to improve their mechanical characteristics and electrical conductivity. Further electrical stimulation (ES) can lead to a substantial increase in expression levels of *S100B*, *GFAP*, *NGFR*, *MBP*, and *MPZ*, which are associated with myelination in SCs and an increase in NGF production, EGF, and hepatocyte growth factor (HGF). This process can also promote differentiation of human adipose-derived mesenchymal stem cells (hADMSCs) into SC-like cells. By constructing a biological scaffold to promote stem cell differentiation into SC-like cells and establishing a milieu favorable to SC-like cell differentiation, the risk to the host from viral transfection or drug induction may be significantly minimized. Li et al. (2021b) demonstrated that using electrostatic spinning, micro-nano processing, and biomaterial surface biologization techniques, the constructed neural regeneration micro-environment scaffold with anisotropic micro-nano composite topology could effectively induce directed SC growth. The Wnt/-catenin, ERK2/MAP, and TGF-pathways upregulate the expression of myelination-related genes and proteins, offering an essential approach to the development of a new generation of functional artificial neural implants.

### Biomaterial and Schwann Cell-Based Engineered Nerve Tissue

The idea of neural tissue engineering focuses on repair of PNI that is different than a single bridging mechanism and leans toward a more biomimetic and bioactive environment that promotes nerve regeneration and axon development. NGCs manufactured using SCs have been demonstrated to stimulate peripheral nerve regeneration. However, as SCs are inaccessible, and cells placed on NGCs are unable to fully cover the catheter surface, long-distance nerve regeneration fails, and motor function recovery becomes compromised. NGCs implanted into bone marrow stromal cells demonstrate more uniform cell dispersion with the assistance of NGF and rotary cell culture systems, which is favorable for establishing a stable 3D bionic environment, enhancing proliferation and differentiation of bone marrow stromal cells into SC-like cells in a substantial manner, and stimulating long-distance peripheral nerve regeneration (Zhou et al., 2020). Georgiou et al. (2015) converted adipose-derived stem cells into an SC cell-like phenotype *in vitro* and then implanted the differentiated cells into type 1 collagen gel, which had been stabilized to produce artificial neural tissue. When the designed nerve tissue generated from ASCs was transplanted into a rat model of sciatic nerve injury, it was shown that this engineered nerve tissue possessed a high capacity for nerve regeneration *in vivo*. Adipose stem cells (ASCs) are a potential cellular vectors because they have the capability of creating cell sheets consisting of cells, intercellular junctions, and extracellular matrix (Yeh et al., 2014). Hsu et al. (2019) prepared functionally enhanced engineered nerves by using ASCs overexpressing BDNF, GDNF, and NGF. This cell plates were shown to be capable of stimulating SC migration, neuron proliferation, and axon development *in vitro* and enhancing the functional recovery, nerves, axons, and myelin sheath regeneration after sciatic nerve injury.

The combination of engineered peripheral nerve tissue with SCs and biomaterials closely mimics the cell composition, extracellular matrix microenvironment, and three-dimensional space structure of the peripheral nerve, resulting in a graft that is closely resembles autologous nerves and has a broad market application potential. Concurrently, as neurological organ technology advances, peripheral nerve tissue engineered from the combination of SCs and biological materials, will also undergo continuous iteration. Not only will this process improve the effect of PNI replacement, but also will help develop a type of peripheral nerve tissue *in vitro* for neurogenesis, repair, and drug screening prior to basic and clinical research. Thibodeau et al. (2022) prepared a new type of tissue-engineered neural catheter. The neural conduit consists of a deactivated hollow tube on the outside and a living fibroblast sheet, which is infused with endothelial cells, rolled into concentric layers on the inside to facilitate the formation of a network containing capillary-like structures for rapid binding to the host neuromicrovascular system after transplantation. When this system in combination with SCs was transplanted into rats, this combination was found to have a higher rates of nerve regeneration than the material-only group.

## Repair of Peripheral Nerve Injury Based on Schwann-Like Cell of New Source

SCs play a critical role in the PNI healing process, but their usage is limited by the difficulty of cell acquisition and its amplification. In addition, isolating, cultivating, and purifying fiber cells, which often multiply quickly in cultured cells, is very challenging (Rutkowski et al., 1995; Andersen et al., 2016; Stratton et al., 2017). To address these obstacles, scientists are investigating safer and more reliable methods for reprogramming various cell sources into induced Schwann-like cells (iSCs) as shown in Table 2.

**TABLE 2:** The methods used in recent years to reprogram cells from different sources into iSCs are summarized.

Original cell	Induction factors	Phenotypic Markers	Animal model	Result	References
BMSCs	BME, RA, FSK, rbFGF, PDGF and HRG	p75,S-100,GFAP and O4	Sciatic nerve injury	The GFP expressing MSCs differentiated into myelin cells and supported the regrowth of nerve fibers within 3 weeks after surgery.	Dezawa et al., 2001
BMSCs	BME, RA, FSK, bFGF, PDGF and HRG	P0 and MAG	Sciatic nerve injury	ISCs-derived artificial grafts have a strong potential to promote peripheral nerve regeneration and can be used to reconstruct long distance gaps in difficult peripheral nerves.	Mimura et al., 2004
BMSCs	BME, RA, FSK, bFGF, PDGF and HRG	PMP22,P0 and MBP	Facial nerve injury	Compared with BMSCs, iSCs provide a faster rate of axon extension and better quality of myelination for peripheral nerve regeneration.	Wang et al., 2011
BMSCs	BME, RA, FSK, bFGF, PDGF and HRG, PROG, insulin and GLUCs.	GFAP, S100B, P0, and PMP22	Sciatic nerve injury	The combined application of PROG, GLUC and insulin significantly improved the differentiation and culture conditions of classical iSCs, and enhanced the stability of morphology, phenotype and functional characteristics of iSCs *in vitro*, as well as the ability of axon growth and endogenous myelin sheath formation *in vivo*.	Liu et al., 2016
ADSCs	engineered substrates with imprinted cell-like topographies	S100b, p75*^NTR^*, and Sox10	–	Specific cell-like topography and associated micromechanical cues can directly differentiate ADSCs into Schwann cells.	Moosazadeh Moghaddam et al., 2019
ADSCs	BME, RA, FSK, bFGF, PDGF and HRG	GFAP, S100 and p75	–	Adipose stem cells can transdifferentiate into iSCs, which may be beneficial for the treatment of peripheral nerve injury.	Kingham et al., 2007
ADSCs	BME, RA, FSK, bFGF, PDGF and HRG	GFAP, S100 and p75	Common peroneal nerve injury	DASCs can be used as a substitute for autologous SCs to form myelin sheaths wrapped with axons *in vivo*, providing nutritional support for axons and promoting peripheral nerve regeneration.	Tomita et al., 2012
ADSCs	BME, RA, FSK, bFGF, PDGF and HRG	S-100, p75 and integrin β4	Sciatic nerve injury	DASCs can accelerate nerve conduction velocity, increase nerve fiber density and myelinated/unmyelinated fiber ratio, and rebuild nerves.	Orbay et al., 2012
ADSCs	BME, RA, FSK, bFGF, PDGF and HRG, PROG, insulin and GLUCs.	S100B, GFAP, PMP22 and P0	Sciatic nerve injury	The combined application of PROG, GLUC and insulin significantly improved the differentiation and culture conditions of classical iSCs, and enhanced the stability of morphology, phenotype and functional characteristics of iSCs *in vitro*, as well as the ability of nerve regeneration and functional recovery *in vivo*.	Kang et al., 2019
MSCs	electrical stimulation by graphene electrodes	p75,α-S100 and α-S100β	–	MSCs can be transdifferentiated into Schwann cell-like phenotypes by electrical stimulation alone without additional chemical growth factors.	Das et al., 2017
UCB-MSCs	BME, RA, FSK, bFGF, PDGF and HRG	GFAP and S-100	–	*In vitro*, UCB-MSCs can differentiate into cells that resemble Schwann cells in morphology, phenotype, and function.	Zhang et al., 2009
hESCs	N2, glutamine, penicillin, streptomycin, bFGF, FSK, NRG1	GFAP, S100, HNK1, P75, MBP and PMP-22	–	HESCs derived neurosphere cells can efficiently differentiate into iSCs with expression of SCs markers.	Ziegler et al., 2011
Human pluripotent stem cells (hPSCs)	N2, B27, BSA, GlutaMAX, BME, CT 99021, SB431542, NRG1, forskolin, RA, PDGF-BB	S100B, NGFR, EGR2, and MPZ	Sciatic nerve injury	Two small molecules SB431542 (a TGF-β inhibitor) and CT99021 (GSK-3 inhibitor) and NRG1 were used and high-quality multipotent SCPs were produced. SCPs can be effectively differentiated into mature SCs with the functions of secreting GDNF, NGF, BDNF and NT-3, which can promote myelination of rat DRG axons *in vitro* and promote axonal regeneration of sciatic nerve injured mice *in vivo*.	Kim et al., 2017
Skin fibroblasts	Lentiviral vectors	Erbb2, Erbb3, Cnx32, Pmp22 and Mpz	–	By driving the expression of two transcription factors, Sox10 and Egr2, human fibroblasts can be successfully transformed into iSCs with unique molecules and functions.	Mazzara et al., 2017
SKIN fibroblasts	Retroviral vectors	S100B	Sciatic nerve injury	Transduction of SOX10 and Krox20 genes directly converts human fibroblasts into functional iSCs. ISCs can form myelin sheath and help mice recover from peripheral nerve injury.	Sowa et al., 2017

*BMSCs, bone marrow mesenchymal cells; BME, beta-mercaptoethanol; RA, retinoic acid; FSK, forskolin; bFGF, basic-fibroblast growth factor PDGF, platelet derived growth factor; HRG, heregulin-beta1; GFP, green fluorescent protein; iSCs, induced Schwann-like cells; PROG, progesterone; GLUCs, glucocorticoids; ADSCs, adipose-derived stem cells; UCB-MSCs, umbilical cord blood-derived mesenchymal stromal cells; hESCs, human embryonic stem cells; NRG1, neuregulin-1; SCs, Schwann cells; MSCs, mesenchymal stem cells; hPSCs, human pluripotent stem cells; GSK-3, glycogen synthase kinase-3; GDNF, glial cell line-derived neurotrophic factor; NGF, nerve growth factor; BDNF, brain-derived neurotrophic factor; NT-3, neurotrophin-3; SCPs, Schwann cell precursors; DRG, dorsal root ganglion.*

Numerous studies have shown that embryonic stem cells (ESCs) and adult stem cells, such as those found in bone marrow, fat, and the umbilical cord, may develop into iSCs (McKenzie et al., 2006; Kingham et al., 2007; Peng et al., 2011; Ziegler et al., 2011; Cai et al., 2017). Although embryonic stem cells proliferate rapidly, their use is controversial and raises ethical questions (Biswas and Hutchins, 2007; Zakrzewski et al., 2019). When ESCs are transplanted into the body, the chance of developing teratomas, localized overgrowth, or cancer increases (Martin et al., 2020). Adult stem cells grow slowly and are often obtained invasively, which limits their therapeutic use (Huang Z. et al., 2020). The development of induced pluripotent stem cells (iPSCs) addresses several constraints associated with stem cells as potential sources of SCs. iPSCs may be isolated from fibroblasts in the skin, peripheral blood mononuclear cells, or even umbilical cord blood cells (Huang et al., 2019). iPSCs have a high capacity for self-renewal and a high rate of proliferation, and they can develop into any type of cell in the endoderm, mesoderm, or ectoderm (Takahashi et al., 2007; Martin et al., 2020). Khuong et al. (2014) utilized lentivirus to promote iSC transformation by driving the expression of two transcription factors, Sox10 and Egr2, in skin fibroblasts. Immunofluorescence research revealed that iSCs, as with primary SCs, exhibited high expression of SC markers, glial fibrillary acidic protein and myelin basic proteins (GFAP and MBP, respectively). *In vitro*, these iSCs form tight myelin sheaths with regular node patterns around axons. Fibroblast growth factors (FGFs) control brain development and cell fate determination during embryonic development by directing cells to differentiate into neurons or glial cells (Mertens et al., 2016). Huang C.W. et al. (2020) seeded adipose-derived stem cells (ADSCs) onto chitosan-coated culture plates, causing the ADSCs to differentiate into a mixed population of neural lineage-like cells (NLCs) after which they then induced NLCs to differentiate into S100β- and GFAP-positive iSCs using FGF9. Wong et al. (2020) compared the effects of different induction times on the proliferation and secretion capacity of iSCs induced by ADSCs and discovered that while the proliferation capacity negatively correlated with induction time, the expression of SC marker proteins S100, MBP, p75, and GFAP positively correlated with induction time. MBP expression resemble normal SCs on day 19 after induction and substantially enhanced axon development compared with early induction. As a result, iSCs appear to be safe to use for 19 days following induction. Dezawa et al. (2001) used beta-mercaptoethanol, retinoic acid, forskolin, basic-FGF (bFGF), platform-derived growth factor (PDGF), and heregulin to induce BMSCs to differentiate into iSCs. The iSCs obtained presented an SC morphology and expressed p75, S100, GFAP, and O4. When retroviruses expressing green fluorescent protein (GFP) were genetically modified and implanted into a sciatic nerve injury model, extensive nerve fiber regeneration and myelination were found. Matsuse et al. (2010) showed that this technique was equally applicable to MSCs from the human umbilical cord. Liu et al. (2012) used conditioned medium to differentiate human hESCs and iPSCs into NCCs and subsequently into SCs from NCCs, establishing the first report on the myelination of human hESCs or iPSC-derived SCs. Subsequently, Kim et al. (2017) converted human iPSCs to self-renewing SCPs by utilizing inhibitors (SB431542 and CT99021) of the TGF-β and GSK-3 signaling pathways, respectively, in addition to using NRG1. Within 1 week, SCPs may differentiate into mature SCs capable of myelination and secreting neurotrophic factors, such as GDNF, NGF, BDNF, and NT-3. Pan and colleagues (Pan et al., 2017) discovered that peripheral blood-derived mesenchymal stem cells (PBMSCs) may generate iSCs that express SC-specific markers (S100, p75NTR, and CNPase) and functional factors (NGF, NT-3, c-fos, and Krox20). This finding solves the problem of the difficult source of tissue engineering seed cells (Schwann cells) after PNI. SCs encapsulating axons in injured sciatic nerves may function well after transplantation. In conclusion, focused modulation of critical molecules in the developmental gene regulatory network lineage of SCs may more effectively stimulate the creation of these various sources of iSCs, indicating a potential therapeutic application.

## Outlook

By describing the process through which SCs repair PNI, our ability to utilize viruses, non-viral vectors, or gene-editing technologies to alter the SC gene may be improved, enabling us to perform additional tasks favorable to peripheral nerve repair. As the safety and control of these methods are enhanced, genetically engineered SCs will have a broader potential for clinical application in peripheral nerve regeneration. The critical element of PNI is the rapid and precise targeting of axons, a process that may be accomplished by developing specialized biological scaffolding for neural bridging. Consequently, cell and biological scaffolding materials may be combined. At this stage, it is critical to develop a biological scaffolding material capable of more accurately simulating the natural milieu of SCs *in vivo*. The source of human SCs severely limits their therapeutic use; however, iSCs offer an excellent solution to this problem. However, an important issue to address throughout the iSC creation process is how to help the iSCs exhibit as many SCs characteristics as feasible while exhibiting as few properties of other cells as possible.

Gene modification can purposefully change the expression of certain genes in SCs thus making it easier to achieve therapeutic goals. However, the corresponding genetic modification also raises more unknown safety issues. After transplantation, SC or iSC survival is low due to the poor microenvironment. Through combinations of biological materials, the internal microenvironment of peripheral nerves was simulated to improve cell survival rate. However, some problems, such as uneven distribution, poor penetration and difficulty in stable release of transplanted cells in biological scaffold materials, exist. Therefore, it is urgent to develop biological scaffold materials that can overcome these shortcomings and achieve industrial mass production, which is of great significance for clinical applications. SC products obtained by tissue and/or genetic engineering and stem cell reprogramming have their own advantages and disadvantages, which can meet the needs of PNI treatment to a certain extent. With the advancement in tissue and genetic engineering, stem cell reprogramming, and other technologies, the purpose of SCs has been extended from simple transplantation to multi-functional complex engineered organogenesis therapeutics thus providing a new perspective on the treatment of PNI and organoid construction.

## Author Contributions

QS, MN, JH, GD, QO, DZ, YD, HH, NL, ZL, PZ, and GL performed the bibliographic research and drafted the manuscript. QS and YD created the table. GD, DZ, and GL prepared the figures. All authors read, approved the final manuscript, analyzed, discussed the literature, commenting on, and approving the manuscript.

## Conflict of Interest

The authors declare that the research was conducted in the absence of any commercial or financial relationships that could be construed as a potential conflict of interest.

## Publisher’s Note

All claims expressed in this article are solely those of the authors and do not necessarily represent those of their affiliated organizations, or those of the publisher, the editors and the reviewers. Any product that may be evaluated in this article, or claim that may be made by its manufacturer, is not guaranteed or endorsed by the publisher.

## Abbreviations

PNI: Peripheral nerve injury
PNS: peripheral nervous system
SCs: Schwann cells
iSCs: induced Schwann-like cells
NCCs: neural crest cells
SCPs: Schwann cell precursors
BL: basal layer
D4: day 4
D7: day 7
lncRNAs: long non-coding RNAs
TGF-β: transforming growth factor-β
NRG1: Neuregulin 1
α BC: AlphaB-crystallin
Notch: Neurogenic locus Notch homolog protein
DRG: dorsal root ganglions
HIF-1α: hypoxia inducible factor − 1 α
VEGF: vascular endothelial growth factor
WD: Wallerian degeneration
NGF: nerve growth factor
AMPK: AMP-activated protein kinase
mTOR: mammalian target of rapamycin
PIGF: placental growth factor
VEGFR1: vascular endothelial growth factor receptor 1
CNTF: Ciliary neurotrophic factor
STAT3: signal transducer and activator of transcription 3
IL-6: interleukin 6
CRISPR: clustered regularly spaced short palindromic repeats
HDAC: Histone deacetylase
Zeb2: Zinc-finger e-box -binding Homeobox 2
HDAC1/2-NuRD: HDAC1 and 2 and nucleosome remodeling and histone deacetylation
NFs: neurotrophic factors
GDNF: glial cell line-derived neurotrophic factor
BDNF: brain-derived neurotrophic factor
LIF: leukemia inhibitory factor
FGF-2: Fibroblast growth factor-2
NT-3: Neurotrophin-3
ANG: acellular nerve graft
COMP: cartilage oligomeric matrix protein
Ang1: angiopoietin1
EGb 761: extract 761
NGC: nerve-guided catheter
PGA: polyglycolic acid
GO: Graphene oxide
MWCNT: multi-walled carbon nanotubes
MSCs: mesenchymal stem cells
TIPS: thermally-induced phase separation
NYs: nanofiber yarns
PPDO: poly(p-dioxanone)
CNT: carbon nanotubes
ES: electrical stimulation
EGF: epidermal growth factor
HGF: hepatocyte growth factor
ADMSCs: adipose-derived mesenchymal stem cells
ESCs: embryonic stem cells
iPSCs: induced pluripotent stem cells
FGFs: Fibroblast growth factors
NLCs: neural lineage-like cells
bFGF: basic-FGF
PDGF: platform-derived growth factor
GFP: green fluorescent protein
PBMSCs: peripheral blood-derived mesenchymal stem cells.
